# Improving Structural, Physical, and Sensitive Properties of Sodium Alginate–Purple Sweet Potato Peel Extracts Indicator Films by Varying Drying Temperature

**DOI:** 10.3390/foods13162477

**Published:** 2024-08-06

**Authors:** Wenxin Li, Mengna Zhao, Xiufang Xia, Yingchun Zhu

**Affiliations:** 1 College of Food Science, Northeast Agricultural University, Harbin 150030, China; 19845916914@163.com (W.L.); zhaomengna98@163.com (M.Z.); 2 College of Food Science and Engineering, Shanxi Agricultural University, Jinzhong 030801, China

**Keywords:** sodium alginate, purple sweet potato peel extracts, indicator films, drying temperature

## Abstract

Sodium alginate (SA)–purple sweet potato peel extracts (PPE) from industrial waste indicator films were developed at different drying temperatures (25, 30, 35, 40, 45, 50, and 55 °C). The effects of drying temperatures on the film’s structural, physical, and sensitive properties were investigated. On the structural properties, scanning electron microscopy, Fourier transform infrared spectroscopy, and X-ray diffraction indicated that compactness, intermolecular interactions, and crystallinity of indicator films were improved at a lower drying temperature. On the physical properties, with the drying temperature increasing, elongation at the break increased significantly (*p* < 0.05); ΔE and water-vapor permeability decreased significantly (*p* < 0.05); and thickness and tensile strength initially increased significantly (90.46 → 98.46, 62.99 → 95.73) and subsequently decreased significantly (98.46 → 71.93, 95.73 → 55.44) (*p* < 0.05), with the maximum values obtained at 30 °C. On sensitivity, the corresponding colors of the films became lighter as the drying temperature increased, and the films exhibited relatively excellent pH and NH_3_ sensitivity, with easily discernible color changes at lower temperatures. The results of this paper revealed that the overall film characteristics are improved at lower drying temperatures, which will provide valuable references for selecting the drying temperature for preparing indicator films as a guide for industrialized production.

## 1. Introduction

Due to growing concerns about food quality and safety, more and more endeavors have been paid to the aspect of monitoring food freshness. Among them, indicator films have attracted much attention due to their metrics of real-time, rapidity, non-destructive, convenience, and naked eye recognition for monitoring protein-rich food freshness, such as livestock [1,2], poultry [3], aquatic products [4,5], and milk [6]. Recently, there has been a significant increase in indicator films made from biodegradable polysaccharides and anthocyanins that are natural, safe, and distinguishable in color changes across a wide pH range [7].

Sodium alginate (SA), a natural linear polysaccharide sourced from the byproducts of brown algae, has been widely used in the preparation of indicator films owing to its outstanding film-forming capacity, edibility, biodegradability, and relatively admirable mechanical properties, especially its almost transparent color that maximizes the display of the indicator color itself [8,9]. As for anthocyanins, they are derived from fruits, vegetables, and flowers and have been frequently used for the development of indicator films, especially for some waste products (residues, peels, etc.) due to their low cost [10,11,12]. Purple sweet potato peel is a by-product of the food industry, with an annual production of 70,000–140,000 tons of potato peels, and exhibits higher anthocyanin content than purple sweet potato flesh, whose anthocyanin is low cost, and has relatively excellent stability. Thus, it has been used in the fabrication of pH indicator films [7,13,14].

The indicator films are usually prepared by casting. As one of the key steps, drying is essential for film formation, a process in which both heat and mass transfer take place. On the one hand, the drying temperature determines the degree of intermolecular reorganization and crystallization of the films, which affects the microstructure, physical properties, and sensitivity. It has been shown that film thickness and moisture content are significantly lower when dried at higher temperatures [15]. Qin et al. [16] prepared Konjac glucomannan/agar films dried at 40, 50, 60, 70, and 80 °C and found that lower temperatures brought about intensive hydrogen-bond interactions and regular structures. This was in a manner similar to that of Liu et al. [17], who prepared chitosan films and noticed a drying process at higher temperatures that led to a lower erratic crystalline network and depressed intermolecular interactions, causing a decreasing tensile strength (TS) and an increasing water-vapor permeability. In contrast, Bhatia et al. [18] fabricated chitosan films/gelatin films dried at 25 and 45 °C. The films dried at 45 °C possessed more crystalline structure, thus exhibiting more transparency and superior mechanical properties. These opposite conclusions could be ascribed to the differences in intermolecular interactions and the microstructure of the biopolymers at different drying temperatures. The pH-indicating properties and NH_3_ sensitivity of anthocyanins are also affected by structural changes in the indicator film [19]. It was reported that films with a loose, porous structure changed color more quickly and reacted more effectively [20]. On the other hand, anthocyanins are active substances of natural origin that are unstable and easily decomposed by heat [21]. Castagnini, Betoret, Betoret, and Fito [22] found that the anthocyanin in blueberry juice was lost by up to 70% in air drying at 50 °C, while it was able to maintain about 50% anthocyanin content at 40 °C. Thus, higher temperatures resulted in a lighter color. Although a mass of studies has been carried out about the effect of drying temperature on the microstructure and physical properties of polysaccharide films [16,17,23,24], few studies have been conducted about the role of the drying temperature on the indicator films’ properties and microstructure.

It is generally believed that SA is more stable below 60 °C, and the degradation rate increases when the temperature exceeds 60 °C. In this study, anthocyanins were first extracted from purple sweet potato peels and then combined with SA by a casting method to prepare indicator films at different drying temperatures (25, 30, 35, 40, 45, 50, and 55 °C). Subsequently, their microstructures were analyzed by scanning electron microscopy (SEM), Fourier transform infrared spectroscopy (FTIR), and X-ray diffraction (XRD). Moreover, the physical properties, barrier properties, and mechanical properties of the films were investigated. Finally, the pH sensitivity and NH_3_ sensitivity of the indicator films were evaluated. This work provides basic data for the selection of drying temperatures for the industrial production of indicator films.

## 2. Materials and Methods

### 2.1. Materials and Reagents

Purple sweet potatoes were obtained from the Furuida store. SA (MW~160 kDa; M/G ratio of approximately 1.5) was purchased from Qingdao Mingyue Seaweed Group Co. LTD (Qingdao, China). All other chemical reagents were obtained from Tianjin Tianli Chemical Reagent Co., LTD (Tianjin, China).

### 2.2. Extraction and Measurement of Anthocyanins

The extraction of purple sweet potato peel extracts (PPE) containing anthocyanins from purple sweet potato peel was slightly modified based on a previous method [25]. The specific peeling process was slightly modified according to industrial processing by referring to the method of Alves et al. [26]. The purple fresh potatoes were cleaned, peeled with a peeler, and dried at 35 °C for 48 h. Subsequently, they were ground and passed through a 60-mesh sieve to obtain a powder. The powder was thoroughly mixed with 80% ethanol (1:20, *w*/*v*) at room temperature for 24 h and then ultrasonicated for 40 min at 400 W at 4 °C. Subsequently, the supernatant was obtained by centrifugation at 5000 r/min for 10 min. The filtrate was extracted by repeating the above process three times. The supernatant was then concentrated by evaporating the ethanol from the supernatant. Finally, the water was removed using a vacuum freeze dryer to obtain PPE. According to the strategy reported by Giusti and Wrolstad [27], with slight modification, the anthocyanin content (AC) was measured in the pH differential way. This was conducted by adding 0.6 g of anthocyanin powder to 100 mL of deionized water, and the solution was subsequently diluted to the appropriate magnification (pH = 1, the absorbance was between 0.2 and 0.7) with 0.025 M KCl (pH = 1.0) and 0.4 M NaAC (pH = 4.5), respectively. The absorbance was measured at 520 nm and 700 nm after 1 h of reaction protected from light. The ACT was determined as mg cyanidin-3-glucoside/g of PPE using Equation (1):

(1) $$\mathrm{ACT}=\frac{\left[\left(A_{520(pH=1.0)}-A_{700(pH=1.0)}\right)-\left(A_{520(pH=4.5)}-A_{700(pH=4.5)}\right)\right]\times M_{w}\times DF\times V\times 1000}{\varepsilon \times L\times W_{t}}$$

where A is the absorbance, M_w_ is the molecular weight of cyanidin-3-glycoside (449.2 g/mol), DF is the dilution factor, V is the original solution volume (mL), ε is the molar extinction coefficient (26,900 L·cm^−1^·mol^−1^), L is the optical path length (1 cm), and W_t_ is the powder weight of the PPE (mg). The ACT in PPE was 513 mg/100 g purple sweet potato peel powder.

### 2.3. Preparation of the Indicator Films

The casting way was used to prepare indicator films according to the method reported by Jiang, Zhang, and Jiang [28] with modifications. First, 1.50 g of SA were added to 100 mL of distilled water. Then, 30% of glycerol (based on the mass of SA) was introduced and stirred until it dissolved entirely. Then, the SA-PPE film-forming solution was obtained by adding 0.6% (*w*/*v*) PPE to the SA solution and stirring until homogeneous. Next, ultrasonication was performed to debubble. Then, 30 mL of the film-forming solution were measured, poured into plastic Petri dishes (8.50 cm × 8.50 cm), and dried using a constant temperature and humidity chamber at different drying temperatures (25, 30, 35, 40, 45, 50, and 55 °C) and 50% relative humidity conditions. Finally, the dried films were cross-linked by immersing them in a 2.00% CaCl_2_ solution. The films were kept at a temperature of 25 °C and a relative humidity of 50% for a duration of 48 h prior to being utilized. The prepared indicator films were labeled SA-PPE25, SA-PPE30, SA-PPE35, SA-PPE40, SA-PPE45, SA-PPE50, and SA-PPE55.

### 2.4. Structural Characterization of Films

#### 2.4.1. SEM

The microstructures of the films were observed by a TM-5570 tungsten filament SEM (Hitachi High-Technologies Corp., Tokyo, Japan) [29]. The film sample planes and cross-sections were fixed on a metal sample stage using double-sided tape, gold sprayed, and the accelerating voltage of 5 kV set, and the magnification of the film surfaces and cross-sections was 1000×.

#### 2.4.2. FTIR

The films were cut to 10 mm × 10 mm, and the PPE powder was scanned using an FTIR (Shimadzu, Kyoto, Japan) in the wave number range of 4000–400 cm^−1^ at a scan rate of 64 s in the wave number range of 4000–400 cm^−1^ using an FTIR (Shimadzu, Japan) to analyze the film composition, as well as the molecular interactions [30].

#### 2.4.3. XRD

The films were cut to 10 mm × 40 mm, and the XRD patterns of the films were recorded using an X’Pert Pro diffractometer (PA Nalytical B.V., Almelo, The Netherlands) equipped with nickel-filtered Cu Kα radiation to analyze the molecular arrangement of the films [31]. The intensity in the spectra was recorded as a function of 2 θ from 5° to 90°, with a step of 0.1°/s. The relative crystallinity degree of the films was calculated using Jade 9.

### 2.5. Physical Properties of Films

#### 2.5.1. Color Properties

The film color features (L*, a*, and b*) were analyzed using a ZE-6000 colorimeter (Nippon Denshoku Industries Co., Ltd., Tokyo, Japan) by a slightly modified method [32]. The total color difference (ΔE) was obtained as stated below:

(2) $$∆E=\sqrt{{\left(L*-L_{0}^{*}\right)}^{2}{{+\left(a*-a_{0}^{*}\right)}^{2}+\left(b*-b_{0}^{*}\right)}^{2}}$$

where L*, a*, and b* are the color values of the indicator films, and L_0_, a_0_, and b_0_ are the values of the SA films, respectively.

#### 2.5.2. Thickness

The film’s thickness was determined according to a recent method [33]. The thickness was recorded at ten random locations on the films using a digital micrometer, and the average value was taken.

#### 2.5.3. Moisture Content and Water Solubility

Moisture content (MC) was determined according to a previous method with slight modifications [34]. The films were cut into squares (20 mm × 20 mm), weighed for initial weight, and then dried at 105 °C until constant weight and weighed for constant weight. The MC was recorded based on Equation (3):

(3) $$MC\;(\%)=(m_{1}-m_{2})/m_{1}\times 100$$

where m_1_ and m_2_ are the initial weight and constant weight of the films, respectively.

Water solubility (WS) was determined according to a previous method, with slight modifications [35]. The films were cut into squares (20 mm × 20 mm), dried at 105 °C until constant weight and weighed for initial constant weight, placed in a centrifuge tube containing 50 mL of distilled water for 24 h, and dried in an oven at 105 °C until constant weight again and weighed for dissolved constant weight. WS was calculated according to the following Equation (4):

(4) $$WS(\%)=\left(m_{3}-m_{4}\right)/m_{3}\times 100$$

where m_3_ and m_4_ are the initial constant weight and dissolved constant weight of the films, respectively.

#### 2.5.4. Mechanical Properties

The mechanical properties, including tensile strength (TS) and elongation at break (EB), were measured based on the method of Zhao et al. [7] using a TA. XT Plus C texture analyzer (Stable Microsystems, Godalming, UK). The films were cut to 10 mm × 50 mm. The EB (%) was documented by computer programs. The TS was obtained by Equation (5):

(5) TS(MPa)=F/(L×d)

where F is the force breaking the films (N), and L and d are the width (mm) and thickness (mm), respectively.

#### 2.5.5. Barrier Properties

##### Water-Vapor Permeability (WVP)

WVP was recorded by the method of Wang et al. [36], with slight modifications. Cut films (20 mm × 20 mm) were adhered to the top of a 2 mL centrifuge tube containing silica gel, placed in a 50 mL centrifuge tube containing saturated potassium sulfate (98% relative humidity), placed at room temperature, and weighed at 1 d intervals. The WVP was calculated as Equation (6):

(6) $$WVP\;(g\cdot m^{-2}s^{-1}{Pa}^{-1})=\left(∆m\times L\right)/\left(∆t\times A\times ∆P\right)$$

where ∆m is the difference in weight of the films (g), ∆t is the time (s), ∆P is the water-vapor partial pressure difference (1754 Pa), L is the film thickness (mm), and A is the effective area of the films (m^2^).

##### Light Transmission of Films

The transmittance of the films was obtained at 290–800 nm using a UV-visible spectrophotometer (T6 Xinshiji, Puxi General Instrument Co., Beijing, China).

### 2.6. Sensitivity of Indicator Films

#### 2.6.1. pH Sensitivity of Indicator Films

The pH sensitivity of the indicator films was implemented based on the methods of Guo et al. [37] and Merz et al. [38] with modifications. The indicator films were submerged in buffer solutions, with pH values ranging from pH 2–13, respectively. The color changes were captured with a camera at 25 °C and under uniform lighting conditions. The color features (L*, a*, and b*) were recorded using Adobe Photoshop 2022. The ΔE was obtained as Equation (2).

#### 2.6.2. Color Response of Indicator Films to Volatile Ammonia

The indicator film response to NH_3_ color was based on that stated by Alizadeh-Sani et al. [25]. An ammonia solution at a concentration of 8 mM was prepared at room temperature, and the films (10 mm × 10 mm) were suspended 10 mm above the solution. Images were taken every 2 min, and the color parameters were accessed using Adobe Photoshop 2022 software. The NH_3_ sensitivity (S_RGB_) was obtained as Formula (7):

(7) $$S_{RGB}=\left(\left|R_{1}-R_{0}\right|+\left|G_{1}-G_{0}\right|+\left|B_{1}-B_{0}\right|\right)/\left(R_{0}+G_{0}+B_{0}\right)\times 100\%$$

where R_0_, G_0_, B_0_, R_1_, G_1_, and B_1_ are the gray values of red, green, and blue of the control films and R_1_, G_1_, and B_1_ are the gray values of red, green, and blue of control the films and the films treated by the ammonia solution, respectively.

### 2.7. Statistical Analysis

The results were expressed as mean ± standard error, and an analysis of variance (ANOVA) was performed on the data using Statistix 8.0 software (Analytical software, St. Paul, MN, USA). Significant differences (*p* < 0.05) were performed by a Tukey test. Plots were performed using OriginPro 2021 (OriginLab, Northampton, MA, USA).

## 3. Results and Discussion

### 3.1. Structural Characterization of Films

#### 3.1.1. Microstructure

**Figure 1:**
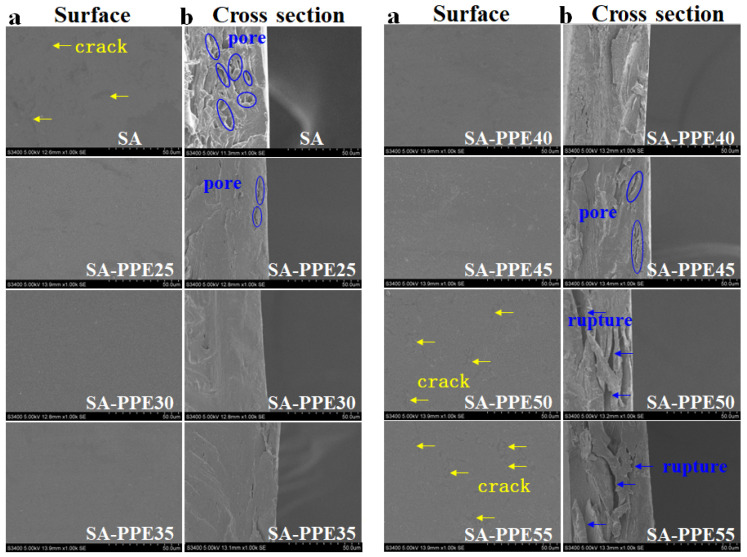
Effect of drying temperature on the structural properties of indicator films. (**a**) SEM images of surface, (**b**) SEM images of cross section of indicator films.

The microstructure of the indicator films is characterized by SEM, which is closely related to the physical properties of the films. The effects of different drying temperatures on the microstructure of the indicator films are shown in Figure 1a,b. The surface of the control SA films was relatively uniform, without folds and bubbles, but there were some small cracks (yellow arrows). The cross-section caused a loose mesh structure with some small pores (blue circles). At lower drying temperatures (25–40 °C), with an increase in drying temperature, the surfaces of the indicator films became gradually smooth and uniform, and the cross-section was denser and more continuous. With the further increase in drying temperature (45–55 °C), cracks appeared on the surface, and pores and ruptures appeared in the cross-section of the indicator films. At lower drying temperatures, the water evaporated slowly, and the drying rate was so slow that more hydrogen bonds were formed between the SA, PPE, and glycerol molecules under sufficient time conditions due to the order entanglement, making the films smoother on the surface and denser in the cross-section [39]. When the drying temperature was too high, the films were dried by evaporating water molecules rapidly directly from the network, forming cavities in the network. In addition, high drying temperatures could also result in increased molecular mobility within the film, disrupting intermolecular hydrogen bonding [25]. Similar phenomena were reported by Zhang et al. [40] in hydroxypropyl methylcellulose/curdlan films that were compared to the films dried at 37 °C. The cross-section of the films prepared at 85 °C was rougher. In contrast, Bagheri, Radi, and Amiri [23] found that the protrusions on the surface of the film gradually decreased and became smoother as the drying temperature increased.

#### 3.1.2. Intermolecular Interactions

**Figure 2:**
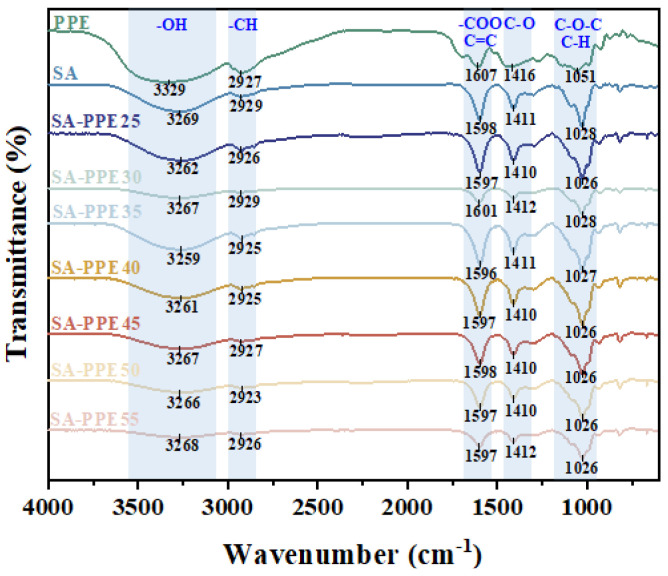
Effect of drying temperature on the FT−IR spectra of indicator films.

The intermolecular interactions between the film matrix molecules are analyzed by the peak intensity and position FTIR map. As indicated in Figure 2, compared to the control SA films, the O–H stretching peak of all the indicator films shifted toward the lower wave numbers. With an increasing drying temperature, the positions of the O–H stretching peaks first shifted toward the lower (blue shift) and then higher (redshift) wave numbers. Meanwhile, the peak intensity first increased and then decreased. After being incorporated with PPE, new hydrogen bonds were formed between the PPE and SA molecules, causing the blue shift of the O–H stretching peaks [7]. When the drying temperature was too low, the molecular movement was so slow that the macromolecules could not fully unfold, limiting the formation of hydrogen bonds. As the drying temperature increased to a suitable temperature, many hydrogen bonds were formed, leading to the blue shift of peak position and increase of peak intensity. With a further increase in the drying temperature, the hydrogen bonds between the matrix molecules were interrupted, resulting from fast molecular motion, causing the redshift of the peak position and the decrease in peak intensity. Qin et al. [16] also reached a similar result with the increasing dry temperature from 40 to 80 °C for konjac glucomannan/agar films. The O–H stretching peaks shifted from 3270 cm^−1^ toward the higher (redshift) wave numbers 3300 cm^−1^, indicating that the hydrogen bonds were broken between konjac glucomannan and agar molecules.

#### 3.1.3. Crystallinity

**Figure 3:**
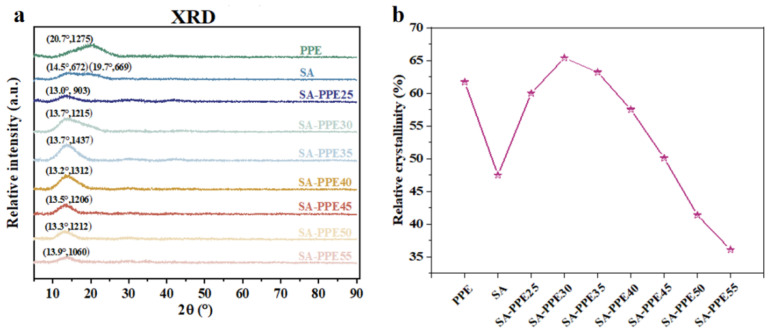
Effect of drying temperature on the XRD spectra of indicator films. (**a**) XRD patterns and (**b**) relative crystallinity of indicator films.

X-ray diffraction patterns can characterize the degree of crystallinity of the films, thus reflecting the regularity of the molecular arrangement of the films, which is closely related to the physical properties of the films (e.g., mechanical properties, optical properties, barrier properties, etc.). As shown in Figure 3a,b, the relative crystallinity of the indicator films increased at lower temperatures after the addition of PPE compared to the control. With an increase in the drying temperature, the relative crystallinity of the indicator films first gradually increased and then gradually decreased, and reached the maximum value of 65.4% at the drying temperature of 30 °C. At a moderate drying temperature, the film matrix molecules had time to recombine to form crystalline regions. While a higher drying temperature was set, the drying rate was faster, and there was not enough time for crystalline regions to form between the film molecules before the water evaporated [41]. Also, the PPE was destroyed and the glycerol was evaporated when the temperature was too high, destroying the crystalline area between them [42,43]. This observation was consistent with that of the previous research reported by Zhang et al. [40], who found that the relative crystallinity of hydroxypropyl methylcellulose/curdlan films dried at 85 °C was lower than the films dried at 37 °C when the curdlan content was ≥ 50%.

### 3.2. Physical Properties of Films

#### 3.2.1. Color Properties

**Figure 4:**
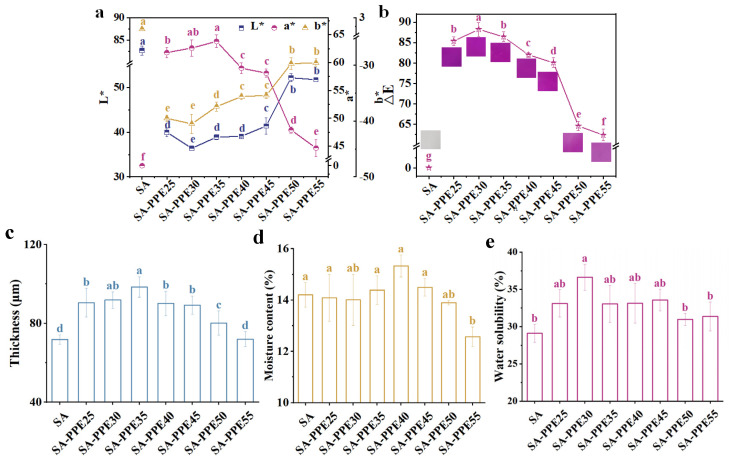
Effect of drying temperature on the color and basic properties of indicator films. (**a**,**b**) Color properties, (**c**) thickness, (**d**) moisture content, and (**e**) water solubility of indicator films. Note: different lowercase letters (a–g) indicate significant differences between different drying temperatures (*p* < 0.05).

The indicator films provide food freshness information by their apparent color, which reflects the preservation of pigments in the indicator films to some extent. As presented in Figure 4a,b, compared to SA films, the L* and b* of indicator films significantly decreased, while a* and ∆E significantly increased (*p* < 0.05). With an increase in the drying temperature, the L* and b* of the indicator films significantly increased, while a* and ∆E significantly decreased (*p* < 0.05), which is in line with the brighter and lighter purple–red appearance color (Figure 4b). The excessive temperature would cause the anthocyanin to undergo a ring-opening reaction [44], causing the methanolic pseudobase structure to change into a chalcone structure, which further generated benzoic acid or aldehydes, destroying the structure of the anthocyanin and causing the PPE indicator and indicator films to become lighter in color [42].

#### 3.2.2. Thickness

Thickness is one of the basic parameters of films, which is closely related to water-vapor barrier properties and mechanical properties. As described in Figure 4c, the thickness obviously rose after PPE was added (*p* < 0.05). As the drying temperature increased, the thickness was first remarkably elevated and then reduced (*p* < 0.05). And the thickness reached a maximum of 98.5 μm at 35 °C. The increase in the thicknesses of the indicator films after the addition of PPE was due to the increase in the amount of dry matter in the film-forming solution. The change in thickness with temperature was related to the evaporation of water and glycerol at high temperatures as well as the thermal degradation of anthocyanins in PPE, which decreased the amount of film-forming material in the film-forming solution and therefore the film thickness decreased. Bagheri, Radi, and Amiri [23] concluded that the decrease in film thickness at higher temperatures was due to the decrease in the mass of glycerol from evaporation and the subsequent decrease in the amount of film-forming material. Bhatia et al. [18] also found that the thickness of chitosan–gelatin–ginger oil blend films dried at a higher drying temperature (45 °C) was lower than the films dried at a lower drying temperature (25 °C).

#### 3.2.3. Moisture Content and Water Solubility

MC is one of the basic parameters of films, which reflects the free volume of water molecules in the film network [45]. As depicted in Figure 4d, the MC of indicator films drastically decreased along with an increase in the drying temperature (*p* < 0.05), and the MC reached a minimum value of 12.6% at 55 °C. Previous researchers had also shown that the MC of hydroxypropyl methylcellulose/curdlan films dried at 85 °C was lower than the films dried at 37 °C, which was attributed to a similar reason for the evaporation of glycerol induced by heat [40].

During transportation, especially when moving from a refrigerated environment to a room-temperature environment, water droplets appear on the package, and the films need to remain stable and undissolved in the presence of water. The WS can reflect the impedance of the films to water. The smaller the WS, the stronger the barrier to water. As shown in Figure 4e, the WS of the indicator films decreased significantly with the increasing drying temperature (*p* < 0.05) and reached a minimum value of 31.0% when the drying temperature was 50 °C. The PPE in the indicator films was a hydrophilic substance due to containing many phenolic hydroxyl groups, and glycerol was a polyhydroxy alcohol that was bound to water through hydrogen bonding [23]. However, high temperatures destroyed the structure of the PPE while glycerol was evaporated. So, the indicator films prepared at high drying temperatures had low WS and good resistance to water, which ensures the integrity of the indicator films in the presence of water. This was also exemplified in the work undertaken by Li et al. [46], who showed that the WS of konjac glucomannan–zein blend films significantly decreased when the drying temperature rose from 40 to 60 °C.

#### 3.2.4. Mechanical Properties

**Figure 5:**
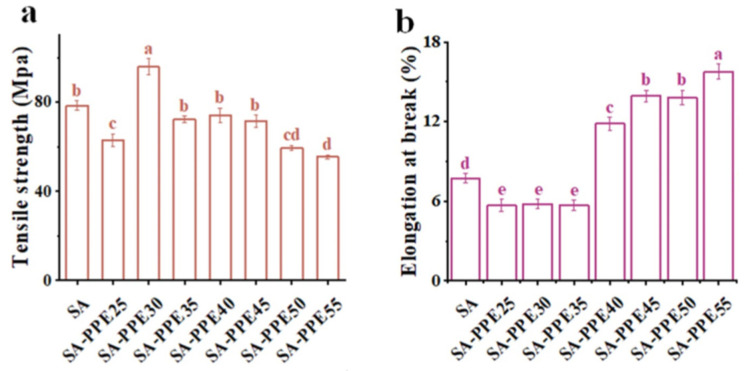
Effect of drying temperature on the mechanical properties of indicator films. (**a**) Tensile strength, (**b**) elongation at break of indicator films. Note: different lowercase letters (a–e) indicate significant differences between different drying temperatures (*p* < 0.05).

Mechanical properties usually include TS and EB, with TS being the maximum ability of the films to withstand externally applied stress and EB being the maximum ability of the films to resist changes in length [47,48]. As shown in Figure 5a,b, the TS and EB of SA-PPE25 films significantly decreased compared to SA films (*p* < 0.05). With an increase in the drying temperature, the TS of the indicator films first drastically increased and then decreased (*p* < 0.05), while the EB remarkably rose (*p* < 0.05), reaching maximum values of 95.7 Mpa and 16.1% for TS and EB at 30 °C and 55 °C, respectively. As far as TS was concerned, the molecules moved slowly, which caused weak intermolecular interactions between the film matrix molecules at too low temperatures (25 °C), causing the reduced TS. As the drying temperature increased, more hydrogen bonds were formed between the macromolecules, making the film network dense and leading to increased TS. When the drying temperature was too high, the film structure collapsed severely, which is in agreement with the SEM results (Figure 1a,b). So, the TS decreased [17]. This was similar to the results of Liu et al. [17], who found that the TS of chitosan was lower at higher temperatures. This was attributed to the fact that the higher the temperature and the faster the drying rate, the more severe the structural collapse of the chitosan film, leading to a reduction in the ordered structure and weakened intermolecular interactions. Anthocyanins in PPE also could be destroyed at high temperatures, making the interaction between SA and PPE molecules in the film network discontinuous, reducing cohesion and reducing TS [18]. Meanwhile, in terms of EB, the increase in EB in the indicator films could be explained by fast-moving molecules, making for greater flexibility. Zhang et al. [40] showed that the elongation of hydroxypropyl methylcellulose/curdlan films dried at high temperatures was higher than that of the corresponding films dried at low temperatures, which was mainly due to the increased compatibility of the films, smoother morphology structure, and reduced crystalline structure at high temperatures.

#### 3.2.5. Barrier Properties

##### Water-Vapor Barrier Properties

**Figure 6:**
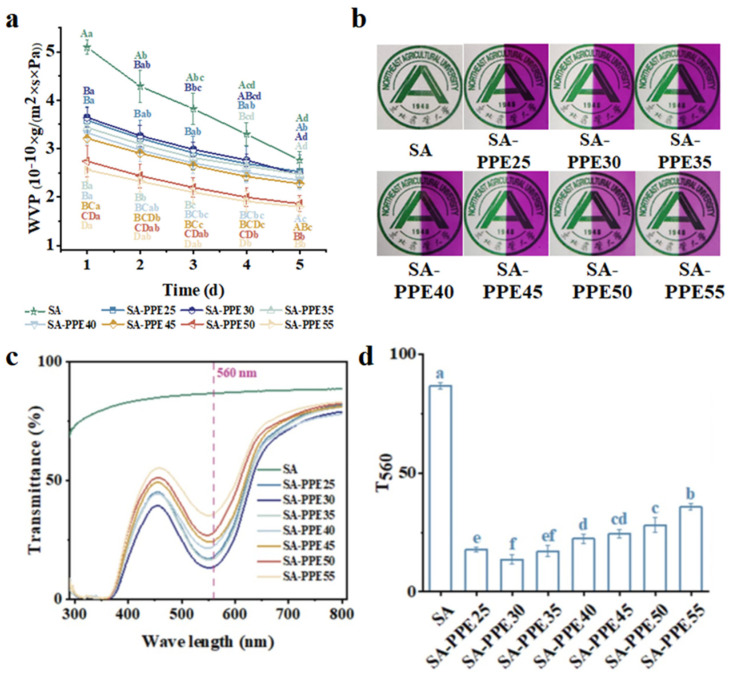
Effect of drying temperature on the barrier properties of indicator films. (**a**) Water-vapor permeability, (**b**) appearance images, (**c**) light transmission, and (**d**) the light transmission at 560 nm of indicator films. Note: for (**a**), different uppercase letters (A–D) indicate significant differences between different drying temperatures (*p* < 0.05), and different lowercase letters (a–d) indicate significant differences between different days (*p* < 0.05). For (**d**), different lowercase letters (a–f) indicate significant differences between different drying temperatures (*p* < 0.05).

Water-vapor barrier properties are reflected by WVP, and the low water-vapor transmission rate resists the dissolution of water into the film and ensures the integrity of the film. As indicated in Figure 6a, the highest WVP was observed for SA films, and the WVP significantly decreased after the PPE was added (*p* < 0.05). Similar findings were reported by Jiang et al. [49], who found that the addition of black-currant extract significantly reduced the WVP of starch/gum ghatti films. The WVP of the indicated films reduced sharply with the increasing drying temperature and storage time (*p* < 0.05). The higher WVP of the SA films was assigned to the lower relative crystallinity (Figure 3b) and looser structure (Figure 1a,b), allowing the water vapor to penetrate. The lower WVP of the indicator films was associated with the lower MC. The MC of the films itself was lower when the indicator films were dried under higher temperature conditions. Therefore, the films reduced the plasticization process under low humidity conditions, preventing the rearrangement of the internal structure and blocking water penetration [50]. Additionally, anthocyanins in PPE containing many hydrophilic hydroxyl groups were thermally degraded at high drying temperatures, thus reducing the number of water molecules that could be bound. Another reason for the reduction in WVP could be that, as the drying temperature increased, the distance the water molecules had to travel through the SA film to permeate increased due to the efficient distribution of PPE [46]. The WVP decreased with the storage time because of the limitation of the packaging environment, which meant that the MC contained in the package increased with the storage time and gradually reached equilibrium, making it difficult for water vapor to enter the tube. Bagheri, Radi, and Amiri [23,41] also reached a similar conclusion that SA films obtained the lowest WVP at higher drying temperatures, which is consistent with the previous study that konjac glucomannan/lemongrass essential oil films had a lower WVP at 50 °C than at 30 °C [41].

##### Light-Barrier Properties

The light-barrier properties are reflected by light transmittance, and the lower the transmittance, the stronger the light-barrier properties. At the same time, the degradation extent of anthocyanin in PPE could be evaluated by the light transmittance at around 560 nm [42]. As seen in Figure 6c, the light transmittance declined after PPE filled the SA matrix. The light transmittance and loss extent of anthocyanin in the PPE first decreased and then increased with the increasing drying temperature, and reached the minimum value at 30 °C, which is in accordance with the apparent color of indicator films shown in Figure 6b. As shown in Figure 6d, the T_560_ also first reduced and then rose with an increasing drying temperature. The decline in the light transmittance of the films added to the PPE was attributed to the result of a certain light absorption by the purple–red PPE. More crystalline areas of the films dried at lower temperatures and enhanced the reflection of light, causing lower light transmittance [51]. The thermal degradation of anthocyanins, in which the anthocyanins were broken down into protocatechuic acid, gallic acid, phloroglucinaldehyde, and so on by heat, caused the lighter color of the indicator films, which reduced the light absorption by the purple–red PPE [42,52,53]. This was also demonstrated in the study carried out by Qin et al. [42], who reported that the light transmittance and degradation extent of anthocyanins in starch/polyvinyl alcohol/Lycium ruthenicum anthocyanins films increased with an increase in temperature.

### 3.3. Sensitivity

#### 3.3.1. pH Sensitivity

The pH sensitivity of indicator films is used to evaluate their potential for monitoring food freshness. As shown in Table 1, when the pH changed from 2 to 13, L* and b* increased sharply in all the indicator films, while a* decreased significantly, meaning the red weakened and the yellow strengthened. All indicator films’ ∆E was greater than five, indicating that the color change of the indicator films could be discerned by the naked eye. At the same pH, the L* of the indicator films increased, while the a* and ∆E decreased significantly, indicating a corresponding lightening of color, which is consistent with the apparent color (Table 1). The change in the color of the indicator films with pH was mainly due to the shift in the structure of the anthocyanins in the PPE, where flavylium cation dominated in acidic conditions, quinoidal base in weakly basic conditions, and chalcone in strongly basic conditions [54]. The ΔE value of the SA-PPE55 was lower than other drying temperatures (25–50 °C) in the pH 2–12 range. The ΔE value of SA-PPE55 showed a significant increase at pH 5–8 compared to SA-PPE35 (38.3 → 77.8, 39.0 → 75.3, 35.9 → 77.6, 33.2 → 76.6, *p* < 0.05). Significant increases also occurred at pH 9, 10, and 12 compared to SA-PPE30 (38.8 → 82.2, 43.0 → 72.0, 37.2 → 69.9, *p* < 0.05). The lighter color of the indicator films with increasing drying temperature was due to the fact that the high temperature destroyed the anthocyanins in the PPE of the indicator films, which reduced the structure of the colored anthocyanins [42]. The above results indicated that the indicator films that were dried at lower temperatures were brightly colored and were easily distinguishable to the naked eye. They had excellent pH sensitivity and, in consequence, had the potential for monitoring meat freshness.

#### 3.3.2. NH_3_ Sensitivity

**Figure 7:**
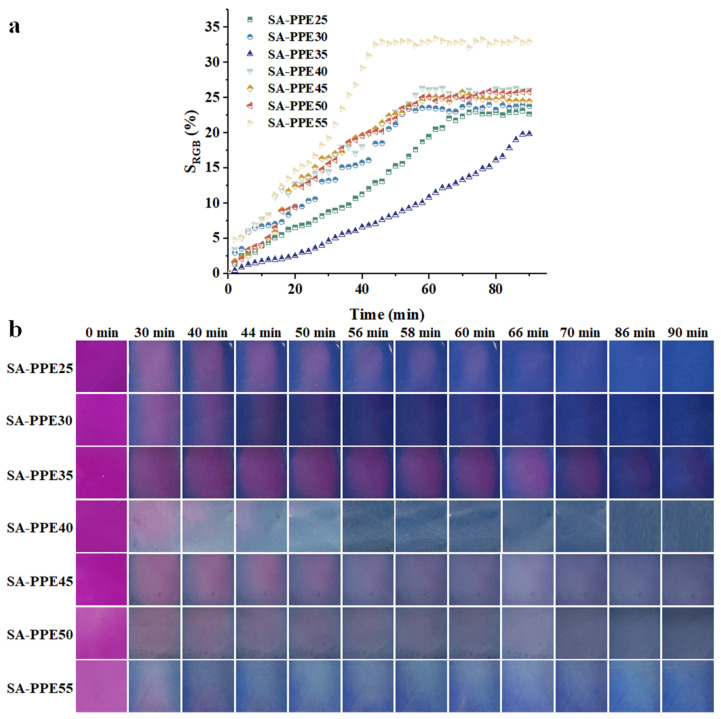
Effect of drying temperature on the NH_3_ sensitivity of indicator films.

The NH_3_ sensitivity of indicator films is also used to assess their potential for monitoring food freshness. S_RGB_ indicated the NH_3_ sensitivity of the indicator films. The larger the S_RGB_, the better the NH_3_ sensitivity. As shown in Figure 7a, as the drying temperature increased, the S_RGB_ of the indicator films increased, and the time required for the color to reach equilibrium was shorter. As depicted in Figure 7b, the color of the indicator films changed when exposed to volatile ammonia as the response time prolonged, with pink → light pink → blue–green. However, the corresponding color was lighter. The color changes of the indicator films with time were attributed to the changes in anthocyanin-related structures in PPE. The anthocyanin structure was transformed from a flavylium cation to a quinonoidal base or chalcone, forming a blue–green color [55]. The color-conversion mechanism of the films is related to the interaction between NH_3_ molecules and the water molecules present on the surface and in the polymer chains, producing OH^−^ species [51]. As the drying temperature increased, the indicator films became more sensitive to discoloration because some of the anthocyanins in the indicator films were destroyed, and fewer water molecules were available to bind. Accordingly, the fewer anthocyanins there were, the less OH^−^ was needed, but the NH_3_ content in the closed container was constant. So, the discoloration was more sensitive. Ma, Du, and Wang [56] investigated the NH_3_ sensitivity of tara gum/polyvinyl alcohol-based indicator films under different relative humidity conditions and found that the higher the relative humidity, the more obvious the color change. This phenomenon was because the higher the relative humidity, the more water molecules attached to the TG/PVA molecules, resulting in a higher production of NH^4+^ and OH^−^, which created a more alkaline environment that changed the structure of the curcumin and, therefore, the faster the color change. This resulted in an increased alkalinity of the environment, which altered the structure of the curcumin and accelerated the color change. This phenomenon occurred because, as relative humidity increased, more water molecules bonded to the TG/PVA molecules, leading to increased production of NH^4+^ and OH^−^ [57]. This result was similar to our study in that the indicator films were all subject to color change by NH_3_ molecules and water molecules. This better sensitivity due to anthocyanin destruction was not expected, and both good sensitivity and anthocyanin integrity should be ensured to prepare the indicator films.

## 4. Conclusions

The effects of drying temperatures (25, 30, 35, 40, 45, 50, and 55 °C) on SA/PPE indicator films were investigated. Excessive drying temperatures resulted in microstructural disruption of the films and weakened physical properties (thickness, MS, and WS) but increased WVP, EB, and NH_3_ sensitivity. At lower drying temperatures (30, 35, and 40 °C), SA/PPE films have excellent TS values, light barrier, crystallinity, and good surface morphology compared to other drying temperatures. This could be due to stronger intermolecular interactions under these conditions. It is recommended to set the drying temperature of SA/PPE films in the range of 30–40 °C to obtain good barrier properties, mechanical properties, pH, and NH_3_ sensitivity. This study provides basic data and theoretical guidance for the rational design of SA-based indicator films. In addition, it is necessary to investigate the effects of other factors in the film fabrication process on the indicator film to achieve continuous industrial production.

## Figures and Tables

**Table 1:** Effect of drying temperature on the pH sensitivity of indicator films.

SA-PPE25	Color	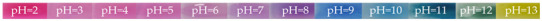											
	L*	43.0 ± 1.2 Ge	53.9 ± 2.1 CDd	56.3 ± 2.0 BCb	49.6 ± 2.3 Fe	57.7 ± 0.8 Bc	52.9 ± 1.2 DEc	40.1 ± 1.5 Hef	51.3 ± 1.4 EFc	36.6 ± 1.1 Ie	34.8 ± 1.6 Ib	51.2 ± 1.8 EFb	65.9 ± 2.2 Ab
	a*	66.2 ± 1.0 Aab	46.5 ± 2.0 Ba	45.8 ± 3.0 Bc	46.9 ± 2.2 Ba	29.6 ± 1.4 Dd	42.0 ± 1.5 Ca	30.3 ± 1.3 Dd	3.00 ± 0.01 Ec	−7.10 ± 0.32 Fc	−11.4 ± 0.5 Ga	−18.1 ± 0.3 Hc	−7.70 ± 0.48 Fc
	b*	−5.10 ± 0.32 Bc	−12.2 ± 0.4 Cd	−12.7 ± 0.5 Cc	−15.6 ± 0.7 Dd	−15.0 ± 0.5 Dc	−23.6 ± 0.5 Fd	−32.5 ± 0.7 Hd	−38.4 ± 1.5 Id	−26.5 ± 1.4 Gc	−21.9 ± 1.4 Ed	−3.90 ± 0.32 Bd	46.3 ± 1.6 Ad
	∆E	85.3 ± 1.0 Aa	64.3 ± 2.5 DEa	62.4 ± 3.5 Ec	68.2 ± 3.0 Cb	51.0 ± 1.5 Hc	65.2 ± 1.8 CDEb	71.8 ± 1.5 Bb	59.3 ± 2.0 Fc	65.2 ± 1.1 CDEb	65.6 ± 1.6 CDc	47.6 ± 1.5 Id	54.3 ± 1.8 Gc
SA-PPE30	Color	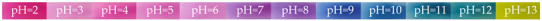											
	L*	46.9 ± 1.2 Dd	57.0 ± 1.2 Bc	51.0 ± 1.1 Cc	57.3 ± 1.7 Bcd	53.6 ± 2.9 Cd	42.7 ± 2.8 Ee	43.0 ± 2.0 Ede	31.2 ± 1.7 Gd	35.3 ± 1.6 Fe	31.0 ± 0.8 Gc	31.0 ± 1.2 Gd	67.7 ± 1.8 Ab
	a*	67.5 ± 0.5 Aa	42.6 ± 0.8 Db	63.0 ± 1.05 Ba	52.2 ± 1.4 Cb	40.8 ± 1.1 Eb	43.3 ± 2.0 Da	33.9 ± 0.7 Fc	12.6 ± 0.7 Ga	−2.30 ± 0.48 Ha	−11.8 ± 0.4 Iab	−25.6 ± 1.1 Je	−2.80 ± 0.42 Ha
	b*	−12.8 ± 0.4 Ce	−16.1 ± 0.6 Df	−14.6 ± 0.5 Dd	−19.6 ± 0.7 Ee	−20.4 ± 0.8 Ee	−34.2 ± 1.2 Gf	−37.7 ± 1.3 He	−48.5 ± 1.4 If	−38.7 ± 0.8 Hf	−24.9 ± 1.9 Fe	−10.8 ± 0.9 Bf	35.9 ± 1.5 Ae
	∆E	84.9 ± 0.9 Aa	60.5 ± 1.1 Gb	79.3 ± 1.2 Ca	68.5 ± 2.0 Fb	62.8 ± 1.7 Gb	77.3 ± 3.4 Ca	73.9 ± 2.0 Dab	82.2 ± 2.2 Ba	72.0 ± 1.2 DEa	70.2 ± 0.8 EFb	69.9 ± 1.3 EFa	44.4 ± 1.2 Hd
SA-PPE35	Color	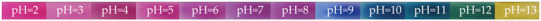											
	L*	48.5 ± 1.5 Ccd	56.8 ± 2.0 Bc	42.3 ± 2.5 DEd	44.9 ± 1.9 Df	43.4 ± 2.9 De	38.4 ± 1.5 Ff	39.3 ± 3.6 EFf	31.8 ± 1.4 Gd	43.4 ± 1.08 Dd	26.7 ± 1.34 Hd	33.5 ± 1.7 Gd	67.1 ± 2.0 Ab
	a*	65.3 ± 0.7 Ab	46.3 ± 2.7 Da	51.3 ± 2.0 Cb	56.1 ± 1.3 Ba	47.8 ± 1.8 Da	43.5 ± 0.5 Ea	43.2 ± 1.9 Ea	7.80 ± 0.42 Fb	−4.10 ± 0.32 Gb	−12.6 ± 0.5 Ib	−22.9 ± 0.7 Jd	−6.30 ± 0.48 Hb
	b*	−9.20 ± 0.42 Bd	−14.2 ± 0.4 Ce	−11.0 ± 0.7 Bb	−15.2 ± 0.4 Cd	−23.4 ± 0.5 Df	−27.3 ± 0.5 Ee	−26.8 ± 0.6 Ec	−44.3 ± 0.5 Ge	−33.2 ± 1.9 Fe	−25.8 ± 0.8 Ee	−9.90 ± 0.57 Be	55.4 ± 4.4 Ac
	∆E	81.7 ± 1.4 Ab	62.8 ± 3.2 Eab	75.4 ± 3.0 BCb	77.8 ± 2.0 BCa	75.3 ± 3.0 BCa	77.6 ± 1.2 BCa	76.6 ± 3.0 BCa	78.6 ± 1.2 ABb	62.3 ± 1.7 Ec	74.6 ± 1.2 Ca	66.5 ± 1.6 Db	61.4 ± 4.1 Eb
SA-PPE40	Color	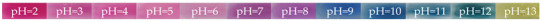											
	L*	48.9 ± 1.0 Ebc	59.7 ± 1.7 Cb	57.0 ± 1.7 Db	55.9 ± 1.0 Dd	63.8 ± 1.2 Ba	44.1 ± 2.1 Ge	46.0 ± 1.6 FGd	54.6 ± 1.3 Db	47.8 ± 2.8 EFc	36.3 ± 1.2 Hb	46.2 ± 1.3 FGc	67.4 ± 2.2 Ab
	a*	62.6 ± 0.8 Ac	38.1 ± 1.3 Dc	47.1 ± 2.3 Bc	41.7 ± 1.3 Cd	34.7 ± 2.3 Ec	37.1 ± 1.4 Db	36.1 ± 2.1 DEb	3.00 ± 0.00 Fc	−7.60 ± 0.52 Gc	−15.5 ± 0.5 Hd	−16.2 ± 0.4 Hb	−7.80 ± 0.42 Gc
	b*	−2.70 ± 0.67 Cb	−12.5 ± 0.9 Dd	−12.2 ± 1.0 Dc	−15.6 ± 0.5 Ed	−16.5 ± 0.5 Ed	−20.7 ± 0.5 Fc	−27.5 ± 0.5 Gc	−38.3 ± 3.1 Id	−30.9 ± 1.6 Hd	−25.7 ± 0.5 Ge	1.10 ± 0.32 Bc	52.2 ± 2.8 Ac
	∆E	78.7 ± 0.9 Ac	54.5 ± 1.9 FGc	62.8 ± 2.7 Cc	60.4 ± 1.5 CDc	50.7 ± 1.8 Hc	67.4 ± 2.5 Bb	68.0 ± 2.4 Bc	56.8 ± 2.7 EFc	57.7 ± 2.9 DEFd	66.4 ± 1.1 Bc	51.4 ± 1.3 GHc	58.6 ± 3.2 DEb
SA-PPE45	Color	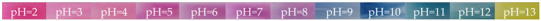											
	L*	48.7 ± 1.5 EFbcd	63.8 ± 1.3 Ba	61.1 ± 2.4 BCa	59.4 ± 1.4 Cc	60.5 ± 0.9 Cb	48.3 ± 4.6 Fd	53.4 ± 2.2 Dc	51.8 ± 1.6 DEc	48.4 ± 1.2 Fc	40.1 ± 2.2 Ga	50.0 ± 2.5 EFb	67.9 ± 1.5 Ab
	a*	60.3 ± 1.1 Ad	37.1 ± 0.6 Cc	38.3 ± 2.5 Cd	41.6 ± 1.4 Bd	31.5 ± 1.2 Dd	29.6 ± 1.0 Ec	27.2 ± 2.1 Fe	−4.40 ± 0.52 Gd	−11.3 ± 0.5 Id	−16.9 ± 0.7 Je	−15.4 ± 0.5 Jb	−7.50 ± 0.85 Hc
	b*	3.10 ± 0.32 Ba	−9.70 ± 0.48 Ec	−6.60 ±0.52 Da	−9.80 ± 0.42 Ec	−10.4 ± 0.5 Eb	−21.4 ± 0.5 GHc	−18.5 ± 0.5 Fb	−22.8 ± 1.0 Hc	−17.5 ± 0.5 Fab	−20.2 ± 0.8 Gc	0.90 ± 0.32 Cc	64.8 ± 3.1 Ab
	∆E	76.9 ± 1.4 Ac	50.5 ± 1.1 FGHd	52.6 ± 3.1 EFd	56.7 ± 2.0 Dd	48.9 ± 1.4 GHc	60.4 ± 3.8 Cc	54.1 ± 2.7 DEd	49.8 ± 1.7 FGHd	51.5 ± 1.0 EFGe	61.3 ± 2.0 Cd	47.5 ± 2.4 Hd	69.6 ± 2.8 Ba
SA-PPE50	Color	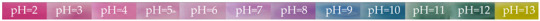											
	L*	50.6 ± 1.4 Gb	61.0 ± 3.0 CDb	60.7 ± 0.8 CDa	62.1 ± 2.1 Cb	65.6 ± 1.4 Ba	60.4 ± 2.8 CDb	59.1 ± 2.2 DEb	56.3 ± 2.5 EFb	54.6 ± 1.1 Fb	35.9 ± 2.0 Hb	58.8 ± 1.6 DEa	73.1 ± 0.9 Aa
	a*	56.8 ± 1.0 Ae	36.3 ± 1.6 Cc	40.4 ± 1.6 Bd	28.8 ± 0.6 De	23.7 ± 1.0 Fe	26.5 ± 2.6 Ed	21.7 ± 1.4 Gf	−7.10 ± 0.32 Hf	−12.7 ± 0.5 Ie	−14.0 ± 0.5 Ic	−16.3 ± 0.7 Jb	−12.6 ± 0.5 Ie
	b*	3.10 ± 0.32 Ba	−4.00 ± 0.00 Ca	−7.20 ± 0.42 Ea	−8.60 ± 0.52 Fb	−8.70 ± 0.48 Fa	−18.4 ± 0.5 Gb	−18.3 ± 0.7 Gb	−18.7 ± 0.7 Gb	−18.7 ± 0.8 Gb	−5.90 ± 0.32 Da	3.00 ± 0.00 Bb	67.0 ± 2.0 Aab
	∆E	73.0 ± 1.2 Ad	50.9 ± 2.4 Dd	54.5 ± 1.6 Cd	45.6 ± 1.8 EFe	39.8 ± 1.3 Gd	48.5 ± 3.5 DEd	46.9 ± 2.6 EFe	44.2 ± 2.4 Fe	46.8 ± 0.9 EFf	61.2 ± 1.9 Bd	39.6 ± 1.6 Ge	70.4 ± 2.0 Aa
SA-PPE55	Color	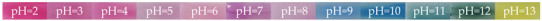											
	L*	54.6 ± 2.0 Fa	63.6 ± 1.08 Ca	55.0 ± 1.4 Fb	66.7 ± 1.5 Ba	64.0 ± 1.5 Ca	65.8 ± 1.0 BCa	67.5 ± 1.7 Ba	60.7 ± 0.8 Da	57.8 ± 1.3 Ea	40.0 ± 1.9 Ga	60.2 ± 2.4 Da	73.7 ± 0.5 Aa
	a*	54.7 ± 2.0 Af	30.7 ± 2.3 Cd	44.6 ± 1.9 Bc	24.1 ± 1.7 Df	20.6 ± 1.6 Ef	16.7 ± 0.5 Fe	15.0 ± 0.7 Fg	−5.90 ± 0.32 Ge	−12.5 ± 0.5 Ie	−17.8 ± 1.0 Jf	−11.2 ± 0.6 HIa	−10.1 ± 0.3 Hd
	b*	2.90 ± 0.32 Ca	−8.30 ± 0.48 Eb	−10.7 ± 0.5 Gb	−4.10 ± 0.32 Da	−8.00 ± 0.00 Ea	−9.20 ± 0.42 Fa	−7.80 ± 0.42 Ea	−15.8 ± 0.4 Ia	−16.4 ± 0.5 Ia	−12.6 ± 0.5 Hb	8.10 ± 0.32 Ba	68.7 ± 1.3 Aa
	∆E	68.9 ± 2.4 Ae	45.7 ± 2.0 Ce	61.9 ± 2.3 Bc	38.3 ± 2.2 EFf	39.0 ± 2.0 Ed	35.9 ± 1.0 FGe	33.2 ± 1.7 Gf	38.8 ± 0.9 Ef	43.0 ± 1.2 Dg	59.4 ± 1.9 Cd	37.2 ± 2.3 EFf	71.5 ± 1.2 Aa

Note: Different uppercase letters (A–J) indicate significant differences between different pH (*p* < 0.05), and different lowercase letters (a–g) indicate significant differences between different drying temperatures (*p* < 0.05).

## Data Availability

The original contributions presented in the study are included in the article, further inquiries can be directed to the corresponding authors.
